# Causes and length of stay of readmission among individuals with traumatic spinal cord injury: a prospective observational cohort study

**DOI:** 10.1038/s41393-023-00874-6

**Published:** 2023-02-25

**Authors:** Marco Franceschini, Lorenzo Cecconi, Jacopo Bonavita, Sanaz Pournajaf, Salvatore Ferro, Maria Cristina Pagliacci, the Italian SCI Study Group, Salvatore Ferro, Maria donate Bellentani, Marco Franceschini, Augusto Cavina, Jacopo Bonavita, Maria Cristina Pagliacci, Annibale Biggeri, Lorenzo Cecconi, Federico De Iure, Giovanni Gordini, Tiziana Redaelli, Maria Vittoria Actis, Giulio Del Popolo, Giannettore Bertagnoni, Renato Avesani, Vincenzo Falabella, M. V. Actis, M. Stillittano, S. Petrozzino, C. Cisari, M. Salvini, T. Redaelli, R. Tosi, C. M. Borghi, A. Bava, C. Pistarini, G. Molinero, A. Signorelli, S. Sandri, F. Simeoni, M. Brambilla, M. A. Banchero, A. Olivero, G. Zanaboni, R. Avesani, G. Bertagnoni, M. Leucci, L. Lain, M. Saia, A. Zampa, P. Del Fabro, M. Saccavini, A. Fanzutto, A. Massone, J. Bonavita, D. Gaddoni, S. Olivi, G. Musumeci, R. Pederzini, H. CerrelBazo, D. Nicolotti, M. Nora, R. Brianti, C. Iaccarino, A. Volpi, A. Lombardi, S. Cavazza, F. Casoni, F. De Iure, G. Gordini, R. Piperno, G. Teodorani, A. Naldi, G. Vergoni, E. Maietti, A. Botti, S. Ferro, G. Pagoto, G. Del Popolo, M. Moresi, M. Postiglione, C. Bini, M. Tagliaferri, M. A. Recchioni, P. Pelaia, L. Di Furia, M. C. Pagliacci, R. Maschke, L. Caruso, L. Speziali, M. Zenzeri, P. Fiore, R. Marvulli, R. Nardulli, C. Lanzillotti, M. Ruccia, M. P. Onesta, T. Di Gregorio, F. Franchina, M. G. Furnari, C. Pilati, M. Merafina, F. Crescia, D. Fletzer, G. Scivoletto, N. Di Lallo

**Affiliations:** 1 Neurorehabilitation Research Laboratory, Department of Neurological and Rehabilitation Sciences, IRCCS San Raffaele, Rome, Italy; 2 San Raffaele University, Rome, Italy; 3 Department of Statistics, Computer Science, Applications “G. Parenti”, University of Florence, Florence, Italy; 4 Rehabilitation Department, Azienda Provinciale Servizi Sanitari APSS, Trento, Italy; 5 Department of Hospital Services, Emilia-Romagna Regional Health Authority, Emilia-Romagna, Italy; 6 Spinal Cord Unit, Santa Maria della Misericordia University Hospital, Perugia, Italy; 7 Age.Na.S., National Agency for Regional Health Services, Department of Hospital Services, Emilia-Romagna Regional Health Authority, Emilia-Romagna, Italy; 8 Department of Neurological and Rehabilitation Sciences, IRCCS San Raffaele Roma, Rome, Italy; 9 Azienda Provinciale Servizi Sanitari APSS, Trento, Italy; 10 SCI Unit, Perugia Hospital, Perugia, Italy; 11 Department of Statistics, University of Florence, Florence, Italy; 12 SCI Surgery Unit and Intensive Care Unit, Maggiore Hospital, Bologna, Italy; 13 SCI Unit, Niguarda Hospital, Milan, Italy; 14 SCI Unit, Città della Salute e della Scienza Hospital, Turin, Italy; 15 SCI Unit, Careggi Hospital, Florence, Italy; 16 SCI Unit, San Bortolo Hospital, Vicenza, Italy; 17 Department of Neurorehabilitation, Don Calabria Hospital, Negrar, Verona, Italy; 18 FAIP, Italian Federation of Paraplegics and Tetraplegics Associations, Rome, Italy; 19 Città della Salute e della Scienza Hospital, Turin, Italy; 20 Alessandria Hospital, Alessandria, Italy; 21 Novara Hospital, Novara, Italy; 22 Piedmont Region, Piedmont, Italy; 23 Niguarda Hospital, Milano, Italy; 24 Pini CTO Hospital, Milano, Italy; 25 IRCCS Maugeri Foundation, Pavia, Italy; 26 Bergamo Hospital, Bergamo, Italy; 27 Legnano - Magenta Hospital, Legnano, Italy; 28 Garbagnate Hospital, Garbagnate Milanese, Italy; 29 Valtellina Hospital, Valtellina, Italy; 30 Lombardy Region, Lombardia, Italy; 31 Don Calabria Hospital, Negrar, Verona, Italy; 32 S. Bortolo Hospital, Vicenza, Italy; 33 Veneto Region, Veneto, Italy; 34 Gervasutta Hospital, Udine, Italy; 35 Udine Hospital, Udine, Italy; 36 Palmanova Hospital, Udine, Italy; 37 Friuli Venezia Giulia Region, Friuli Venezia Giulia, Italy; 38 Santa Corona Hospital, Pietra Ligure, Savona Italy; 39 Montecatone Rehabilitation Institute, Imola, Bologna Italy; 40 Villanova d’Arda Hospital, Piacenza, Italy; 41 Parma Hospital, Parma, Italy; 42 Baggiovara Hospital, Modena, Italy; 43 Maggiore Hospital, Bologna, Italy; 44 Cesena Hospital, Cesena, Italy; 45 Ferrara Hospital, Ferrara, Italy; 46 Emilia-Romagna Region, Emilia-Romagna, Italy; 47 Careggi Hospital, Florence, Italy; 48 Tuscany Region, Tuscany, Italy; 49 Ospedali Riuniti, Ancona, Italy; 50 Marche Region, Marche, Italy; 51 Perugia Hospital, Perugia, Italy; 52 Umbria Region, Umbria, Italy; 53 Bari Hospital, Bari, Italy; 54 IRCCS Maugeri Foundation, Cassano Murge, Bari, Italy; 55 IRCCS Maugeri Foundation, Ceglie Messapica, Brindisi, Italy; 56 Puglia Region, Puglia, Italy; 57 Cannizzaro Hospital, Catania, Italy; 58 Villa Sofia Hospital, Palermo, Italy; 59 Sicily Region, Sicily, Italy; 60 Alesini CTO Hospital, Rome, Italy; 61 Ostia Hospital, Rome, Italy; 62 IRCCS S. Lucia Foundation, Rome, Italy; 63 Lazio Region, Lazio, Italy

**Keywords:** Rehabilitation, Rehabilitation

## Abstract

**Background:**

Secondary conditions may reduce function and participation in individuals with chronic Spinal Cord Injury (SCI). The knowledge of reasons for readmission to the hospital may be enlightening to prevent them and remodel the health services.

**Study design:**

Multicenter prospective observational study of all consecutive readmissions of persons with SCI after rehabilitation completion.

**Objectives:**

To explore the characteristics of individuals with SCI readmitted to the hospital, the reasons for readmissions and the burden on hospitalization in terms of length of stay (LoS) for different conditions.

**Setting:**

31 Italian specialized SCI centers.

**Methods:**

Data on people with traumatic SCI readmitted to SCI centers were recorded about: age, sex, SCI level and severity group, geographical origin, readmission causes, clinical interventions during hospitalization, LoS and discharge destination. Linear and multiple logistic regression analyses were performed considering LoS (days) as dependent variable for correlations with independent variables. All tests were two-sided.

**Results:**

Among 1039 persons with traumatic SCI enrolled (mean age 46, males 85%, tetraplegia 43%), 59.09% of the readmissions were caused by urological problems, 39.74% by pressure injury and 35.41% by spasticity (68% readmitted for ≥2 causes, associated with longer LoS). The mean LoS was 48 days: pressure injury, rehabilitative needs, sexual, bowel, and pain problems were associated with longer and urological problems with shorter LoS. People from the South of the country were frequently (68%) readmitted to the northern centers.

**Conclusions:**

Urological problems, pressure injury and spasticity were the most frequent causes of re-hospitalization in individuals with traumatic SCI. The migration trend seeking SCI-specific treatments suggests geographic areas to which health care organizations need to pay more attention.

## Introduction

Spinal cord injury (SCI) is a complex condition and people with SCI often experience serious secondary medical and non-medical problems that impact on their vulnerability enough to be readmitted to the hospital [1]. Furthermore, a wide range of secondary conditions may reduce their function, community participation, quality of life, and may involve high costs [2, 3]. In recent years, the increase in the age of incidence and in life expectancy [4, 5] have increased interest in SCI complications and chronic secondary conditions and their consequences. Several of these can cause readmission to health facilities, with important personal, social, and economic repercussions.

Recent studies have investigated the health care utilization after SCI from different points of view, as well as readmission rates and its burden, especially in the first period at home after discharge from rehabilitation [6, 7]. Reasons for readmissions following SCI have also been reported and have proved to be quite different in several studies [8, 9]. These studies have identified urological, skin, and respiratory problems as the main causes of readmission. From the literature we know that older age and more severe neurological injuries (quadriplegia and complete injuries) are predictors of hospital readmission for secondary conditions [10–12]. Also, a part of the SCI population with functional, mobility and social limitations may be less easily reached by follow-up programs and more exposed to risk of readmission shortly after rehabilitation discharge [13]. The only data about the Italian situation regarding readmissions after SCI are described in a survey, carried out 20 years ago in a network of Italian rehabilitation SCI-specialized centers [14]. Readmissions were found for approximately the same absolute number of admissions for acute traumatic and non-traumatic SCI in the study period. The main burden, in terms of total days of stay, was represented by functional gain programs, pressure injuries, and urological complications.

There are typically two types of readmission in Italy: general medicine acute wards that deal primarily with medical complications (i.e., urological with surgical necessities, plastic surgery, serious infections, pneumonia, etc.) or specialized SCI rehabilitation centers that deal primarily with rehabilitation-related problems and clinical consequences (such as spasticity, pain, respiratory, urological or bowel management problems, pressure injury in postoperative phase, etc.).

Age and life expectancy trends certainly influence characteristics and burden of readmissions after SCI in a chronic phase. As a result of the multifaceted characteristics of readmissions and the differences in SCI-related health services across countries, it is difficult to examine this topic.

No studies have been conducted in Italy after 2008. Studies conducted in other countries are not uniform regarding readmission rates and causes, depending on local, demographic, injury-related conditions, as well as study settings and design [8–10].

A better understanding of numerous lifelong necessities of persons with SCI who experience a hospital readmission could be beneficial for public health facilities. Consequently, longitudinal planning of SCI health services in Italy could benefit from this knowledge regarding prevention and/or treatment of long-term SCI complications. The current prospective observational cohort study has been designed to detect the characteristics of persons with SCI readmitted to hospital at least 1 year after a period of post-acute rehabilitation program, the reasons for hospitalization and hospital burden, in terms of length of stay (LoS) for different conditions in order to provide an updated overview on this data to the public health services and to better face the lifetime needs of individuals with SCI.

## Methods

**Fig. 1:**
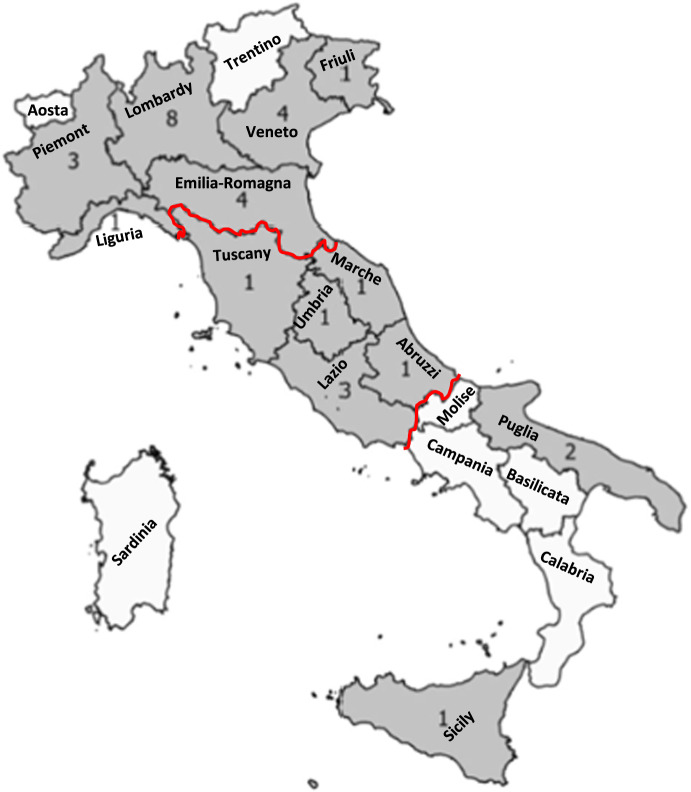
Italian regions and the number of centers participating in the study. The red line indicates the subdivision of three geographical macroregions of Italy: Northern, Central, and Southern Italy.

This prospective observational cohort study was conducted by the Italian Spinal Cord Injury (SCI) study Group, involving 31 specialized SCI centers from 13 Italian regions (Fig. 1) [15]. The study was approved and obtained a grant from the Italian Ministry of Health (CCM number 15756 of 17-07-2012 and “Institutional research”). A regional network for a systematic data collection was created under the supervision of National Agency for Regional Health Services (Age.Na.S.).

All consecutive readmissions of persons with SCI (traumatic or non-traumatic etiology) for any clinical or rehabilitation necessity and their relative LoS, from February 1st 2013 to October 31st 2015 were recorded in an electronic database. The physician of each participating center responsible for the research project prospectively collected the data at admission (T1) and discharge (T2) over the re-hospitalization period. People who were still hospitalized at the end of enrollment (October 31st, 2015) were assessed at discharge regardless of the date. Readmission was defined as re-hospitalization of individuals with SCI who had completed the post-acute rehabilitation program, at least 12 months before readmission.

In this article, only data on readmissions of people with traumatic SCI (TSCI) were considered. Exclusion criteria were readmission LoS < 72 h; age < 16 years; non-traumatic etiology; and other neurological conditions leading to SCI (congenital e.g., spina bifida, new SCI in the context of palliative care, neurodegenerative disorders e.g., Multiple sclerosis, amyotrophic lateral sclerosis and Guillain Barré syndrome).

The patients’ demographic characteristics included in this analysis were: sex, age, and geographical origin of the patient. In light of the fact that in Italy the specialized care facilities for people with SCI are not homogeneously distributed throughout the country, we have also collected information on the geographical origin of the inpatient’s residence, grouped in North, Center, and South as well as the region of the facility where they were readmitted. This data tracked and highlighted the “migration” of individuals with SCI for receiving SCI-specific healthcare services among regions. It was also registered whether they were readmitted from home to the SCI centers or transferred from other facilities. In particular, this data was collected to observe how many readmissions were related to the transfers from acute general medicine wards, since normally, in Italy persons with SCI are readmitted to general medicine acute wards and rarely to SCI specialized units. Following the treatment of their urgent clinical cause of readmission, they can be transferred to the SCI specialized centers for rehabilitation. At T1 the following clinical data were collected: level and severity group of injury defined by International Standards for Neurological Classification of SCI (ISNCSCI) and ASIA Impairment Scale (AIS), respectively. For the statistical analysis, level and severity grade of injury were grouped as: AIS ABC-paraplegia (T1-S5 ABC); AIS ABC-tetraplegia (C1-C4 ABC, C5-C8 ABC); and all D [16]. Causes of readmission were defined as urological, pressure injury(s), respiratory, musculoskeletal, pain, spasticity, bowel, and sexual problems. Complex clinical evaluation (in terms of evaluating complex and personalized assistive devices), rehabilitation training, and social and/or nursing needs impacting home care were also recorded. In many readmission episodes more than one cause of readmission was recorded explaining the number of causes exceeding the number of readmissions. All causes were analyzed separately. For statistical analysis, a dichotomous variable was considered, defined as 1 or 2 or more reasons for readmission. Furthermore, in addition to the cause recorded at readmission, the type of clinical interventions performed during hospitalization was also recorded. This included urologic, pressure injury, pain, spasticity, bowel, and sexual problems. It was also recorded whether any surgical intervention (invasive or minimally invasive; e.g., plastic surgery for pressure injury or botulinum toxin injection by cystoscopy for spasticity) was performed. If mechanical ventilation was initiated for respiratory problems, this was recorded as a noninvasive procedure, except in the case of a tracheostomy. A dichotomous variable was also used for the interventions recorded, defined as 1 intervention versus 2 or more interventions. At T2, in addition to the LoS readmission, the discharge destination was also collected, meant as patient returning home or referral to social and health care facilities (acute general medicine ward, nursing home, rehabilitation center, and specialized SCI center).

The main outcome considered as dependent variable was LoS (days) and it was analyzed for correlation with independent variables.

### Statistical analysis

Categorical variables were calculated as frequencies and percentages for the whole sample. Standard descriptive statistics were used to summarize data, with respect to demographic and clinical characteristics.

Linear regression was used to investigate which factors were associated with LoS. Independent variables of interest were readmission from (home, acute general medicine ward, nursing home, rehabilitation center, and specialized SCI center), sex, age, migration, severity group, interventions and causes of readmission. Multiple logistic regression analyses were performed to account for several confounding variables simultaneously and included all variables of interest.

Analysis was performed by using STATA version 12.1 (StataCorp. 2011. Stata Statistical Software: Release 12. College Station, TX:StataCorp LP).

All tests were two-sided, and an alpha level of 0.05 was set for statistical significance.

## Results

Out of the 1827 total consecutive readmissions in the 31 SCI-specialized centers during the study period, 1039 met the inclusion criteria. The remaining were excluded for the following reasons: non traumatic etiology of SCI (*n* = 524), stay for less than 72 h (*n* = 228), missing data including LoS or cause of admission (*n* = 36).

The mean age (±SD) at readmission was 46 (±15) years. The characteristics of the people with SCI who underwent to readmission are reported in Table 1: males were involved in 85% of cases (male/female ratio 5,7:1); according to the level of the lesion, 43% presented tetraplegia and 54% paraplegia. At T1 complete lesions resulting in complete motor impairment, AIS A and AIS B were the most prevalent (65% and 14%, respectively). When grouping patients by level and SCI severity grade by AIS classification, 12% were included in C1–C4 ABC group, 27% in C5–C8 ABC, 49% in T1-S5 ABC, and 7% in all D lesions.

**Table 1:** Descriptive analysis of people with SCI in readmission episodes.

Variable	Category	*n*	%
Sex	Female	158	15%
	Male	881	85%
Neurological level	Paraplegia	558	54%
	Tetraplegia	447	43%
	_missing	34	3%
ASIA Impairment	A	678	65%
	B	144	14%
	C	109	10%
	D	70	7%
	_missing	38	4%
Severity group	C1-C4 ABC	130	12%
	C5-C8 ABC	277	27%
	T1-S5 ABC	512	49%
	all D	70	7%
	_missing	50	5%
Readmitted from	Home	788	76%
	Acute gen. med. ward	131	13%
	Nursing home	13	1%
	Rehabilitation	46	4%
	SCI Rehab. Center	8	1%
	_missing	53	5%
Destination at discharge	Home	985	95%
	Acute gen. med. ward	21	2%
	Nursing home	13	1%
	Rehabilitation	18	2%
	SCI Rehab. Center	2	0%

As for most of readmissions, 76% of persons accessed from their home at admission, the remainder had a previous admission to an acute general medicine ward (13%), or to a specialized SCI center in a different geographical area or other sources (11%). Expectedly, most part of the participants were discharged to home (95%); the small remainder to acute general medicine (2%), nursing home (1%), and other rehabilitation centers (2%). In 542 of readmissions, people lived in the north of Italy, while 210 were resident in Center, and 277 in South and Italian islands.

Two hundred ninety-six readmissions (28%) involved people with traumatic SCI readmitted in rehabilitation centers located in a different region than people residence/geographical origin at admission. This “health migration” mainly occurred from southern regions to the North and Center or, in part, from Center to North. Indeed, one in three individuals with SCI readmitted to the facilities located in North were resident in another geographical area (Table 2).

**Table 2:** Health migration across the Italian geographical areas.

	Readmission area			
Geographical origin at admission	Central regions	Northern regions	Southern regions	
Central regions	142	62	6	210
Northern regions	8	534	0	542
Southern regions	31	189	57	277
				1029

(Central/Northern/Southern regions of Italy); 10 missing not considered (3 persons with residence out of Italy, 7 missing residence).

**Fig. 2:**
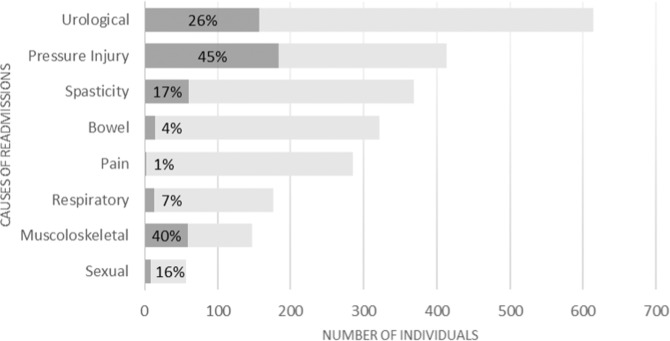
Causes of readmission including clinical complications, and respective impact of interventions during the hospital stay. The clinical conditions and the respective impact of interventions are shown in gray and black, respectively.

The most frequent clinical complications recorded at T1 were urologic problems (*n* = 614), pressure injuries (*n* = 413), and spasticity (*n* = 368). Invasive interventions were performed to treat the corresponding complication in 160 (26%), 186 (45%), and 63 (17%) of these cases, respectively. In a relevant number of readmissions other three conditions were found: new rehabilitative needs (rehabilitation training in 424 readmissions), long-term complex clinical evaluation (in 375), social and/or nursing needs impacting on home care (in 126). The description of the clinical complications causes of readmission in the centers and the clinical interventions carried out during stay is presented in Fig. 2.

In 311 readmissions (30%), only 1 cause was recorded, in 703 (68%) 2 or more causes were registered (in 25, 2%, missing). In 382 readmissions, patients underwent 0 or 1 clinical intervention, in 573, 2 or more interventions were offered (in 84, 8%, missing).

The LoS median (range IQ) was 29 days (12–64).

When analyzing LoS as a function of the independent variables, univariate analysis (Table 3*P* value) showed that transfer to a location other than residence (eg, to a general acute care unit or another center SCI) was associated with an increase in LoS of 45 days; residence in a region other than the location of the rehabilitation center (approximately 7 days more), classification in the “C5–C8 and T1-S5” group (approximately 4 days). There was a direct association between some readmission causes and LoS, which were significantly associated with it: social care needs (32 days longer LoS), pressure injuries (29 days), rehabilitation problems (21 days), sexual problems (18 days), bowel problems (14 days), and pain (8 days). Conversely, urological causes of readmission were significantly associated with shorter LoS (−16 days). As for clinical intervention, a significant association was found between longer LoS and having received interventions for pressure injury both invasive (i.e., surgical operation; 40 days LoS after surgery) and non invasive (i.e., accurate injury medications, mobilization, correct positioning, and other usual care during the hospitalization; 21 days); respiratory surgical intervention (tracheostomy; 31 days), bowel non-invasive intervention (14 days) and having more than 1 cause or needing more than 1 intervention during the admission period. In contrast, urological intervention, both invasive and non-invasive, was associated with shorter LoS (−34 and −9 days, respectively).

**Table 3:** Univariate and multivariate analysis of LoS against independent variables.

Variabile	Categoria	Beta (more days of stay)	*P* value	Multi-Beta	Multi-*P* value
Readmitted from	Home				
	Other	45.0	**<0.001**	45.7	**<0.001**
Sex	Female				
	Male	1.13	0.794	1.15	0.789
Age		0.063	0.530	0.18	0.081
Migration (Nordth/Center/South)	No				
	Yes	6.7	0.051	11.7	**0.001**
Severity group	C1–C4 ABC				
	C5–C8 ABC	3.3	0.540	8.5	0.104
	T1-S5 ABC	3.5	0.475	8.1	0.091
	all D	−4.9	0.509	−5.3	0.466
Intervention number	1				
	>1	12.7	**<0.001**	0.03	0.994
Interventions	Urological	−9.4	**0.004**		
	Urological Invasive	−34.8	**<0.001**		
	Pressure Injury	20.8	**<0.001**		
	Pressure Injury invasive	39.6	**<0.001**		
	Pain	8.1	**0.021**		
	Pain invasive	17.7	0.541		
	Spasticity	3.2	0.348		
	Spasticity invasive	−2.2	0.739		
	Bowel	14.2	**<0.001**		
	Bowel invasive	2.2	0.871		
	Sexual	15.4	**0.037**		
	Sexual invasive	29.5	0.078		
	Respiratory	−2.1	0.623		
	Respiratory invasive	30.7	**0.028**		
	Muscoloskeletal	10.8	0.054		
	Muscoloskeletal invasive	3.0	0.659		
Causes Number	1				
	>1	15.2	**<0.001**	15.8	**0.003**
Cause	Complex evaluation	7.9	**0.014**		
	Urological	−15.9	**<0.001**		
	Pressure Injury	29.2	**<0.001**		
	Muscoloskeletal	7.6	0.086		
	Respiratory	0.3	0.939		
	Pain	8.2	**0.019**		
	Spasticity	2.3	0.473		
	Bowel	13.7	**<0.001**		
	Sexual	17.6	**0.01**		
	Rehabilitation	20.8	**<0.001**		

*P* value significance <0.05 ;Beta (days longer LoS). Significant *p*-values are reported in bold.

In the multivariate analysis (Table 3) coming from other places rather than home (i.e., acute general medicine ward or different SCI centers or other); coming from another region with respect to the rehabilitation center’s location, not being classified in the group “all D” and recording >1 cause of readmission were all independently associated with increased LoS.

## Discussion

The aim of this SCI centers-based study was to explore the characteristics of people living with SCI who needed readmission, as well as the causes and main interventions implemented in 13 regions of Italy, in order to identify possible points of criticism in a network of SCI centers. This multicenter study, involving 31 Italian centers specialized in the comprehensive treatment of SCI and rehabilitation, included more than one thousand consecutive readmissions after traumatic SCI (representing 44% of all patients treated during this period). It shows that secondary complications in persons with SCI occur long after the acute trauma. In a study conducted in Italy between 1997 and 1999, including both traumatic and non-traumatic SCI, readmissions accounted for 51.0% of the total admissions (*n* = 2070) registered during the 2-year study [14]. The results highlighted the value of this type of longitudinal data collection and comparative studies on the health status of the chronic SCI population in our country.

The results of this current study revealed that the most frequent clinical complications were urological problems (60%), of these 25% treated through invasive surgery; pressure injury (40%) surgically treated in almost half of the cases; and spasticity reported in one third of readmissions, (17% managed by invasive surgery). These results confirm the data reported by various studies on principal reasons for hospital readmission following a period of rehabilitation [8, 9, 17–20]. All reports included skin-related problems (e.g., pressure injury) and the genitourinary system (e.g., urinary tract infections [UTIs]) and complications of the upper urinary tract as the most frequent reasons for readmission. Some of the aforementioned clinical complications demonstrated in this study require specific treatment options (e.g., intrathecal baclofen or botulinum toxin) that are available only in specific centers.

Multivariate analysis on LoS showed that being classified in the three most serious neurological classifications (not D group), readmitted from facilities other than home, and readmitted in SCI centers located in regions other than the residency area was independently predictive of longer LoS. The comprehension of the most frequent secondary complications leading to readmission in more severe SCI may help to develop preventive strategies [21].

The long LoS was independently correlated with receiving assistance far from home due to the inability to find skilled health care services nearby the residency, linked also to the low prevalence of SCI among neurological conditions. The last point has already been demonstrated in acute care management [22]. Networks with precise geographical referrals can, however, assist in avoiding delays in taking care of secondary conditions and complications [23].

In the analysis of the geographic areas of readmission with respect to the area of origin for “health migration”, people from the south of Italy tend to move to centers located in the north (68%). Despite improvements in the health service network for people with SCI in Italy over the last few decades, the supply and demand for health services remain unbalanced. Accordingly, the National Health Service must be encouraged to better organize its healthcare network for individuals with SCI to cover the whole territory.

The data analysis allows an update of the general framework on any possible condition contributing to readmission, in order to avoid under-reporting the wide range of needs after SCI as suggested by principally qualitative evaluations [24] thus enlightening the main necessities. In more than 40% of the sample, a rehabilitation request was recorded among causes of readmission. This finding is consistent with the data found by Middleton et al. (11%) [18] and Skelton et al (12%) [7]. It mirrors also the characteristic of the Italian network of specialized SCI centers, which are mainly categorized as rehabilitation centers.

In a proportion of cases (12%), social needs were registered at admission. There was a direct significant correlation between this readmission cause and LoS causing a heavy burden (additional 32 days of stay) on the health care system. This aspect was also pointed out by research incorporating both medical and non-medical variables. Dryden et al. reported that multidisciplinary outreach services have a role to play in providing information and support for many aspects of not only medical but also social care for persons with SCI [25].

Providing adequate facilities and attention to pressure injuries is essential. Literature reports its impact as consequential when considering the LoS associated with this specific complication [8, 14, 18]. The median of LoS, considered as proxy of this burden, was 29 days in line with what was found by Pagliacci et al. (median = 28.5) [14], but not comparable with other studies [8, 18–20]. Maybe longer LoS found in this current study was due to the SCI center-based nature of the study that did not include minor and simplest cases. The LoS shows direct correlation with causes of readmission; in fact pressure injury is associated with increase in LoS, when treated by both conservative (21 days) and surgical methods (40 days after surgery). Pressure injury represents still a serious condition, affecting individuals with SCI with alarming consequences on their quality of life [26].

177 cases of readmission were attributed to respiratory complications, with 13,7% of cases requiring invasive surgical operations (such as tracheotomy and subsequent mechanical ventilation). This small but significant rate may be justified by the growing skill of SCI dedicated centers to manage persons with SCI suffering from high tetraplegia and respiratory failure.

According to our results, a large proportion of people with traumatic SCI had tetraplegia (40% with cervical ABC injuries). This may indicate a higher risk of complications and health care and/or rehabilitation needs in this more frail population [27], as identified also by non-neurological factors in comparison to individuals with paraplegia and incomplete D injuries [17, 28].

As a final note, 85% of the patients who were readmitted were male. Data presented here do not reflect general epidemiological rates, but rather the Italian reality. In fact, epidemiological data in Italy show a higher incidence of men with traumatic SCI in the last decades compared to other international countries [15]. However, due to the different objectives of the study, we were unable to determine whether sex can be considered a risk factor.

Some limitation of this study should be considered when interpreting the findings. The study design did not allow to explore the entire burden of readmission of a prevalent population or a cohort of persons with SCI and it was not possible to compare results of this study to others SCI population-based studies. Furthermore, it is to consider that a part of patients can be missed who were admitted to acute general medicine wards for complications and were not transferred to SCI centers. Besides, longitudinal SCI population-based studies of readmissions report that a part of people with long term needs after SCI may not have had access to specialized SCI centers. Indeed, in a sample of Individuals with SCI in Great Britain between 1990 and 1996, 64% required hospital treatment for late medical complications (127 patients, with 481 readmissions), but only 58% were readmitted into specialized SCI centers [8]. Finally, there is a long time between data collection and manuscript submission due to the long period between the completion of data collection, their extraction and analysis, and the numerous group meetings for discussion of the results and consensus on the concept for the manuscript preparation.

In this study, readmissions shorter than 72 h were not considered since, in Italy, they are usually linked to non-complicated issues and did not reflect the study objectives. This implies that a high number of readmissions caused by urological problems (data not included), such as urological minimally invasive surgery, which usually does not require hospitalization, may have been ruled out. All the readmissions considered for this study are referred to people who had completed the rehabilitation of the acute SCI and had been discharged at least 1 year before the readmission. Only SCI readmissions among the network of specialized SCI centers were considered, hence missing cases with exclusively mild rehabilitation and/or minor medical needs.

This remains an important study that sheds light on readmissions and complications among individuals with Traumatic SCI in Italy that will hopefully inform care, policy, and further studies to improve long-term outcomes.

## Supplementary information

Data Set 1

## Data Availability

The data supporting the findings of this study are available as supplementary data.
